# Association of Medicaid coverage with emergency department utilization after self-harm in Korea: A nationwide registry-based study

**DOI:** 10.1371/journal.pone.0306047

**Published:** 2024-06-25

**Authors:** Ga In Han, Sikyoung Jeong, Insoo Kim, Min Ah Yuh, Seon Hee Woo, Sungyoup Hong

**Affiliations:** 1 Department of Emergency Medicine, Daejeon St Mary’s Hospital, College of Medicine, The Catholic University of Korea, Seoul, Republic of Korea; 2 Department of Emergency Medicine, Incheon St Mary’s Hospital, College of Medicine, The Catholic University of Korea, Seoul, Republic of Korea; Dayeh University, TAIWAN

## Abstract

**Background:**

Self-harm presents an important public health challenge. It imposes a notable burden on the utilization of emergency department (ED) services and medical expenses from patients and family. The Medicaid system is vital in providing financial support for individuals who struggle with medical expenses. This study explored the association of Medicaid coverage with ED visits following incidents of self-harm, utilizing nationwide ED surveillance data in Korea.

**Methods:**

Data of all patients older than 14 years who presented to EDs following incidents of self-harm irrespective of intention to end their life, including cases of self-poisoning, were gathered from the National ED Information System (NEDIS). The annual self-harm visit rate (SHVR) per 100,000 people was calculated for each province and a generalized linear model analysis was conducted, with SHVR as a dependent variable and factors related to Medicaid coverage as independent variables.

**Results:**

A 1% increase in Medicaid enrollment rate was linked to a significant decrease of 14% in SHVR. Each additional 1,000 Korean Won of Medicaid spending per enrollee was correlated with a 1% reduction in SHVR. However, an increase in Medicaid visits per enrollee and an extension of Medicaid coverage days were associated with an increase in SHVR. SHVR exhibited a stronger associated with parameters of Medicaid coverage in adolescents and young adults than in older adult population.

**Conclusion:**

Expansion of Medicaid coverage coupled with careful monitoring of shifts in Medicaid utilization patterns can mitigate ED overloading by reducing visits related to self-harm.

## Introduction

More than 700,000 deaths were caused by suicide worldwide in 2019, making suicide the 17th most prevalent cause of death [1]. In Korea, suicide ranks as the fifth most common cause of death, with the highest incidence occurring among nations belonging to the Organization for Economic Cooperation and Development (OECD) [2]. A previous study has reported that people in the 15–29 and 70–79 age groups have the highest suicide mortality rates [3].

Self-harm is defined as deliberate, nonfatal self-injury or self-poisoning irrespective of the apparent suicidal intent [4]. Self-harm is a risk factor for suicide. Patients who are hospitalized after self-harm demonstrate a suicide rate that is 37 times higher than that in the general population [5]. It has been reported that the rate of emergency department (ED) visits after self-harm is the highest during teenage years [6, 7] and that self-harm may carry a higher risk of recurrence and result in completed suicide in older adults [8, 9]. Additionally, when we look at self-harm by gender, women are more likely to harm themselves than men. However, completed suicide is more common in men than in women [10].

Suicide rates fluctuate depending on economic variables such as economic cycles, unemployment rates, economic growth and the overall level of economic development [11–13]. An earlier study has highlighted that a significant barrier to accessing mental health care, especially for individuals with lower incomes, is the absence of insurance coverage [14]. Medicaid is a government insurance program that provides comprehensive coverage of healthcare to low-income individuals who are unable to pay medical costs [15].

In Korea, the medical benefit (Medicaid) system was introduced in 1977. Since then, it serves as a medical safety net for low-income people having health risks, guaranteeing their health rights. Implementation of the National Basic Living Protection Act was a response to the 1997 financial crisis, during which South Korea experienced notable increases in unemployment rates and poverty levels, leading to a continuous expansion of Medicaid enrollees [16]. Eligibility criteria for Medicaid enrollees include those with an income recognition amount below 40% of median income, domestically adopted children, homeless, refugees, and others. As of November 2022, number of Medicaid enrollees was about 1.52 million, accounting for 3.8% of the general population in Korea. Annual Medicaid expenses have reached 67.2 billion dollars [17].

Previous studies have indicated that the expansion of Medicaid is linked to enhancements of mental healthcare services [18, 19] and, self-assessed overall and mental well-being [20]. Other studies have demonstrated that Medicaid expansion has a beneficial impact, leading to a reduction in suicide rates [21–23]. Nevertheless, these studies faced limitations due to their reliance on annual telephone surveys, the utilization of a before-and-after study design and the absence of quantified independent variables. In addition, such research studies were conducted exclusively within the United States for those with only private insurance without a nationwide conventional health insurance coverage.

Therefore, this study aimed to investigate quantitative associations between Medicaid coverage-related indexes and ED utilization after self-harm on a general population over 14 years old. In addition, this study investigated whether these associations held for adolescents and young adults (AYA) aged 15–34 years with the highest self-harm rate and older adults over 64 years with the highest mortality and recurrence rates following self-harm. This research presents findings from a comprehensive nationwide longitudinal analysis of ED surveillance data in Korea which has comprehensive national health insurance.

## Materials and methods

### Data acquisition

Across 16 provinces in Korea, the public has unrestricted access to 410 EDs. The Korean National Emergency Medical Center (NEMC) oversees the Korean National Emergency Medical Information System (NEDIS), a system that collects data on 67 demographic and clinical variables from patients who seek medical attention at EDs. We obtained panel data from NEDIS, covering patients over 14 years old treated at EDs for self-inflicted injuries or poisonings irrespective of their motives or the severity of their suicidal intentions between January 2014 and December 2019. The NEDIS database encompassed details such as age, gender, onset time, self-harm method, route to reach ED and mental status of patients upon arriving at ED. Assessment of altered mental status was done with the Alert, Verbal, Pain, and Unresponsive (AVPU) scale. Information pertaining to the population categorized by age and gender for each was sourced from the Korean Statistical Information System (KOSIS) administered by Statistics Korea (https://kosis.kr/index/index.do). We gathered variables related to Medicaid coverage, which encompassed statistics such as number of Medicaid enrollees, government Medicaid spending, total count of Medicaid visits (including both hospitalizations and outpatient visits) and number of Medicaid coverage days, all of which were sourced from KOSIS. We also collected socioeconomic indicators from KOSIS for each province, including employment rate, disposable income and crime rate.

### Variables

Self-harm identification took place in the ED entry’s triage room by registered nurse inquired about intentional injury infliction from patients or those who accompanied patients. For unconscious patients, the determination was based on accounts of guardians or emergency medical technicians who evaluated the patient on site. The identification of self-harm was recorded through a checkbox, which was then promptly sent to and stored on the NEDIS server. Medicaid enrollment rate was calculated by dividing the total number of Medicaid enrollees by the mid-population number for each province. Medicaid spending per enrollee, Medicaid visits per enrollee, and coverage days per enrollee were calculated by dividing crude values with mid-population for each province and each year.

### Measurement and standardization of outcome variable

The primary outcome of this study was ED self-harm visit rate (SHVR) per 100,000 population calculated as the annual number of ED visits after self-harm divided by the mid-population for each province. ED SHVR was calculated for the general population older than 14 years of age as well as for AYA (aged 15–34 years), middle-aged adults (aged 35–65 years) and older adults (aged 65 and over) for each province.

### Statistical analysis

General demographic characteristics of self-harm patients were compared using chi-square analysis for discrete variables. However, the chi-square test is known to be sensitive to sample size (e.g., more than 200 participants), often leading to rejection of the null hypothesis of a statistically significant difference. Therefore, Cramer’s V was used to measure the strength of association between variables with chi-square analysis. Cramer’s V value was categorized as follows: values above 0.25, very strong association; values between 0.15 and 0.25, strong association; values ranging from 0.10 to 0.15, moderate association; values between 0.05 and 0.10, weak association; and values from 0.00 to 0.05, no or very weak association [24].

A multivariate regression analysis was performed on a general population over 14 years old and two specific age groups using SHVR as a dependent variable without considering interaction effects. The regression model included Medicaid enrollment rate, Medicaid spending, Medicaid visits, and Medicaid coverage days per enrollee as independent variables. The primary outcome measure of interest was SHVR, which quantified the frequency of self-harm incidents adjusted for the population number involved in a specific age group in each province. SHVR assumes a Poisson distribution since incidents of self-harm are very rare (S1 Fig). A generalized linear model (GLM) with a Poisson distribution and log-link function was used to test the significance of association between SHVR and the independent variable. When the regression analysis was conducted, socioeconomic factors (crude income per capita, employment rate and crime rate for each province) were set as control variables and analyzed. After conducting these analyzes, visit rate ratio (VRR) and its confidence interval (CI) are presented. We employed R-squared as a statistical parameter to quantify the extent to which independent variables in a GLM account for the variability observed in a dependent variable. Collinearity among independent variables was evaluated using variance inflation factor (VIF). R statistical program version 4.2.2 (R Foundation for Statistical Computing, Vienna, Austria) was employed for all statistical analyzes.

### Ethics approval and patient consent statement

This study protocol has been approved by the institutional review board of Daejeon St Mary’s Hospital, The Catholic University of Korea (No DC23ZISE0083). This study is registry-based and the NEDIS database was completely anonymized, so informed consent was waived.

## Results

### Demographics and characteristics of subjects by main exposure

Summary information on Medicaid enrollment and coverage throughout the study duration is provided in S1 Table. The rate of Medicaid enrollment consistently fell from 3.8% to 3.3% throughout the study period. Concurrently, the count of Medicaid visits and coverage days consistently increased. Characteristics and distribution of self-harm patients aged 14 and over who visited ED during the study period are described in S1 Text, S2 and S3 Tables.

Characteristics of self-harm patients categorized based on Medicaid enrollment rate intervals are presented in Table 1. Cross-analysis between patient age group distribution and Medicaid enrollment rates did not show a significant association (V = 0.061; p < 0.001). Medicaid enrollment rate also demonstrated a very weak association with the distribution of gender of self-harm patients (V = 0.030; *p* < 0.001), self-harm method (V = 0.047; p < 0.001), and mental status of patients upon arrival at ED (V = 0.026; p < 0.001), indicating that the Medicaid enrollment rate did not depend on general characteristic variables of self-harm patients.

**Table 1:** Demographic characteristics of self-harm patients according to the main exposure factor (Medicaid enrollment rate).

Variable		Medicaid enrollment rate (%)				(Cramer’s V) P-value
		0–2	2–4	4–6	Over 6	
Age, n (%)	15–24	9,839 (21.4)	15,148 (20.8)	5,869 (17.0)	386 (15.3)	(0.061) <0.001
	25–34	8,456 (18.4)	13,355 (18.3)	5,158 (15.0)	403 (16.0)	
	35–44	9,033 (19.7)	13,897 (19.1)	6,336 (18.4)	523 (20.8)	
	45–54	8,776 (19.1)	13,110 (18.0)	6,734 (19.5)	525 (20.9)	
	55–64	4,898 (10.7)	7,912 (10.9)	4,322 (12.5)	287 (11.4)	
	65–74	2,517 (5.5)	4,449 (6.1)	2,704 (7.8)	187 (7.4)	
	75–84	1,856 (4.0)	3946 (5.4)	2,641 (7.7)	161 (6.4)	
	85–94	495 (1.1)	993 (1.4)	643 (1.9)	39 (1.6)	
	95-	33 (0.1)	46 (0.1)	42 (0.1)	4 (0.2)	
Gender, n (%)	Female	25,458 (55.5)	39,586 (54.3)	17,759 (51.6)	1,262 (50.2)	(0.030) <0.001
	Male	20,445 (44.5)	33,270 (45.7)	16,690 (48.4)	1,253 (49.8)	
Self-harm method, n (%)	Poisoning	25,935 (56.5)	41,418 (56.8)	21,860 (63.5)	1,566 (62.3)	(0.047) <0.001
	Stabbing	10,843 (23.6)	17,068 (23.4)	6,653 (19.3)	447 (17.8)	
	Hanging	3,330 (7.3)	5,044 (6.9)	2,575 (7.5)	194 (7.7)	
	Struck	2,306 (5.0)	2,988 (4.1)	943 (2.7)	104 (4.1)	
	Fall	1,411 (3.1)	1961 (2.7)	900 (2.6)	49 (1.9)	
	Submersion	332 (0.7)	901 (1.2)	342 (1.0)	22 (0.9)	
	Burn	103 (0.2)	228 (0.3)	94 (0.3)	13 (0.5)	
	TA	146 (0.3)	225 (0.3)	124 (0.4)	6 (0.2)	
	Machine	5 (0.0)	35 (0.0)	14 (0.0)	7 (0.3)	
	other	1,492 (3.3)	2,988 (4.1)	944 (2.7)	107 (4.3)	
Mental status, n (%)	Alert	30,607 (66.7)	48,388 (66.4)	21,206 (61.6)	1,615 (64.4)	(0.026) <0.001
	Verbal	7,239 (15.8)	11,872 (16.3)	6,493 (18.9)	381 (15.1)	
	Pain	4,700 (10.2)	7,474 (10.3)	3,921 (11.4)	272 (10.8)	
	Unresponsive	3,357 (7.3)	5,121 (7.0)	2,827 (8.2)	247 (9.8)	
Total		45,903	72,856	34,449	2,515	

### Results of generalized linear model analysis

**Fig 1:**
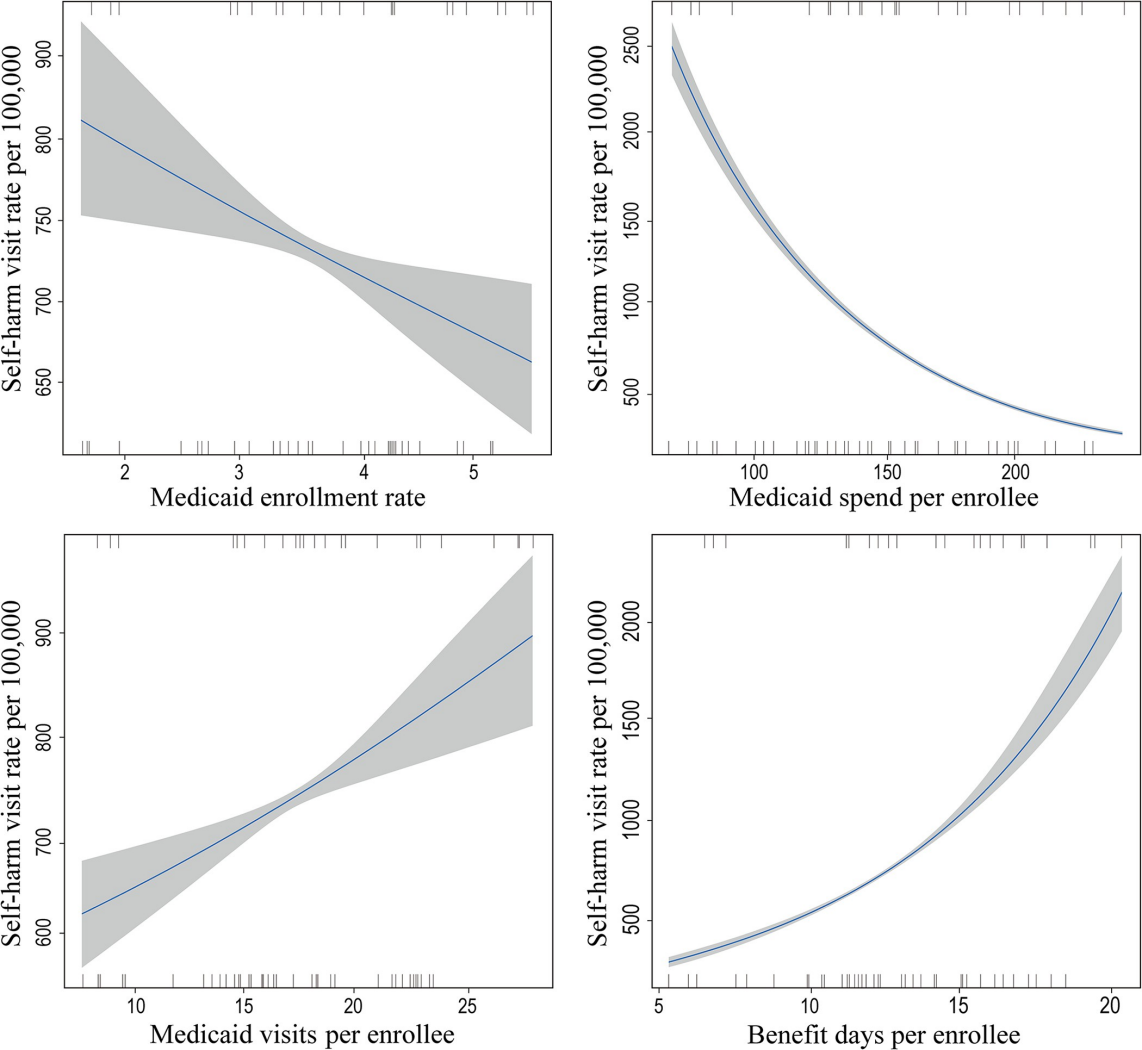
Regression plots (blue line) with 95% confidence intervals (gray).

Fig 1 depicts polynomial regression plots for SHVR of the general population with Medicaid coverage variables. SHVR decreased as Medicaid enrollment rate and Medicaid spending per enrollee increased in general population older than 14 years. There was a noteworthy reduction of 14% in SHVR associated with a mere 1% increase of Medicaid enrollment rate (p < 0.001, Table 2). Likewise, a rise of 1,000 Korean Won in Medicaid spending per enrollee was connected to a 1% decrease in SHVR (p < 0.001). Conversely, SHVR increased when the number Medicaid visits and Medicaid coverage days per enrollee climbed up (p < 0.001 and p < 0.001). SHVR in the male adults showed the same tendency. However, in female adults, SHVR was found to be significantly associated with the count of Medicaid visits and Medicaid coverage days (p < 0.001 and p < 0.001).

**Table 2:** Summary of generalized linear model analysis for Medicaid coverage factors predicting emergency department self-harm visit rate in the general population older than 14 years.

Age group (R-squared)	Factor	VRR	95% CI	P-value
General population (0.455)	Medicaid enrollment rate	0.86	0.83–0.89	< 0.001
	Medicaid spending per enrollee	0.99	0.98–0.99	< 0.001
	Medicaid visits per enrollee	1.19	1.09–1.30	< 0.001
	Coverage days per enrollee	1.18	1.17–1.20	< 0.001
Male population (0.534)	Medicaid enrollment rate	0.76	0.73–0.78	< 0.001
	Medicaid spending per enrollee	0.98	0.98–0.98	< 0.001
	Medicaid visits per enrollee	1.34	1.23–1.47	< 0.001
	Coverage days per enrollee	1.21	1.20–1.23	< 0.001
Female population (0.380)	Medicaid enrollment rate	0.96	0.94–0.999	< 0.001
	Medicaid spending per enrollee	0.99	0.99–0.99	< 0.001
	Medicaid visits per enrollee	1.05	0.96–1.15	0.277
	Coverage days per enrollee	1.15	1.14–1.17	< 0.001

VRR; visit rate ratio, CI; confidence interval.

A significant increase in SHVR with an increase in Medicaid enrollment rate was observed exclusively in older adult males (p < 0.001, Table 3). A decrease of Medicaid spending per enrollee was associated with an increase of SHVR across the entire cohort of older adults as well as in both male and female older adults (p < 0.001, p < 0.001, and p = 0.045, respectively). Increases of Medicaid visits per enrollee and coverage days per enrollee also were associated with an increase of SHVR (all p < 0.001).

**Table 3:** Summary of generalized linear model analysis for Medicaid coverage factors predicting emergency department self-harm visit rate in older adults (> 65 years old).

Age group (R-squared)	Factor	VRR	95% CI	P-value
Older adults (0.464)	Medicaid enrollment rate	1.02	0.98–1.14	0.056
	Medicaid spending per enrollee	0.99	0.99 – 0.99	<0.001
	Medicaid visits per enrollee	1.38	1.25 – 1.52	<0.001
	Coverage days per enrollee	1.09	1.07 – 1.10	<0.001
Male older adults (0.478)	Medicaid enrollment rate	1.15	1.11–1.18	<0.001
	Medicaid spending per enrollee	0.99	0.98 – 0.99	<0.001
	Medicaid visits per enrollee	1.32	1.23–1.32	<0.001
	Coverage days per enrollee	1.08	1.06–1.09	<0.001
Female older adults (0.463)	Medicaid enrollment rate	1.03	0.98–1.05	0.250
	Medicaid spending per enrollee	0.99	0.98 – 0.99	0.045
	Medicaid visits per enrollee	1.43	1.36–1.48	<0.001
	Coverage days per enrollee	1.10	1.08–1.12	<0.001

VRR; visit rate ratio, CI; confidence interval.

In the middle-aged group of 35–64 years old, an increase in Medicaid coverage days per enrollee was positively associated with an increase in SHVR (Table 4). This linear relationship was statistically significant (p < 0.001). However, other variables analyzed did not demonstrate a statistically significant level of association with SHVR.

**Table 4:** Summary of generalized linear model analysis for Medicaid coverage factors predicting emergency department self-harm visit rate in middle-aged adults (35–64 years old).

Age group (R-squared)	Factor	VRR	95% CI	P-value
General middle-aged adults (0.478)	Medicaid enrollment rate	0.82	0.75–0.90	0.045
	Medicaid spending per enrollee	0.99	0.96–1.02	0.314
	Medicaid visits per enrollee	1.06	0.99–1.14	0.071
	Coverage days per enrollee	1.24	1.18–1.30	< 0.001
Male middle-aged adults (0.499)	Medicaid enrollment rate	0.98	0.97–0.99	0.105
	Medicaid spending per enrollee	0.99	0.99–1.01	0.151
	Medicaid visits per enrollee	1.15	1.11–1.22	< 0.001
	Coverage days per enrollee	1.01	1.00–1.03	< 0.001
Female middle-aged adults (0.423)	Medicaid enrollment rate	0.99	0.97–1.02	0.156
	Medicaid spending per enrollee	1.01	0.99–1.03	0.126
	Medicaid visits per enrollee	1.34	1.15–1.05	0.277
	Coverage days per enrollee	1.32	1.21–1.41	< 0.001

VRR; visit rate ratio, CI; confidence interval.

In AYA aged 15–34, SHVR was more closely associated with parameters of Medicaid coverage (Table 5). SHVR decreased when both Medicaid enrollment rate and Medicaid spending per enrollee were increased in the general population older than 14 years as well as in male and female subgroups (all p < 0.001). Conversely, there was a significant increase in SHVR following an increase of Medicaid visits or an increase in Medicaid coverage days per enrollee (all p < 0.001).

**Table 5:** Summary of generalized linear model analysis for Medicaid coverage factors predicting emergency department self-harm visit rate in adolescent and young adults aged 15–34 (AYA).

Age group (R-squared)	Factor	VRR	95% CI	P-value
AYA (0.650)	Medicaid enrollment rate	0.69	0.65–0.73	<0.001
	Medicaid spending per enrollee	0.98	0.98–0.99	<0.001
	Medicaid visits per enrollee	1.28	1.16–1.39	<0.001
	Coverage days per enrollee	1.38	1.37–1.40	<0.001
Male AYA (0.737)	Medicaid enrollment rate	0.79	0.74–0.81	<0.001
	Medicaid spending per enrollee	0.98	0.98 – 0.99	<0.001
	Medicaid visits per enrollee	1.38	1.36–1.39	<0.001
	Coverage days per enrollee	1.33	1.32–1.35	<0.001
Female AYA (0.534)	Medicaid enrollment rate	0.76	0.58–0.86	<0.001
	Medicaid spending per enrollee	0.99	0.98 – 0.99	<0.001
	Medicaid visits per enrollee	1.11	1.04–1.27	<0.001
	Coverage days per enrollee	1.42	1.40–1.43	<0.001

VRR; visit rate ratio, CI; confidence interval.

R-squared value is a parameter that quantifies the extent to which the variability in a dependent variable can be explained by the variability in independent variables. When we analyzed regression results for the general age adults, we found that the R-squared value was 0.455. Interestingly, the R-squared value was higher in the male subgroup at 0.534 compared to that in the female subgroup at 0.380. The same pattern was observed across older, middle-aged, and AYA. There were no notable collinearity issues observed among independent variables in any regression models (all VIF < 2.548, S4 Table).

## Discussion

The main goal of this research was to investigate the connection between Medicaid coverage factors and the utilization of ED services at the provincial level. We observed a reduction in ED visits following self-harm incidents by both AYA and older adults when Medicaid coverage, including factors such as Medicaid enrollment rate and Medicaid spending per enrollee, was expanded.

In this study, the primary outcome variable (SHVR) was the ED visit rate associated with self-harming, a significant risk factor responsible for suicidal behavior [25]. No prior research has examined the association between Medicaid coverage and risk of self-harm. Nevertheless, numerous previous studies have focused on suicide. Thus, we conducted a literature review on the relationship between suicide and Medicaid expansion. The Patient Protection and Affordable Care Act (ACA) had broadened Medicaid coverage in 2014 to all adults with income at or below 138% of federal poverty level in 26 states including the District Columbia. It was well noted that the expansion of Medicaid had resulted in a reduction of 1,818 suicide cases at a rate of -0.40 (95% CI: -0.66 to -0.14) per 100,000 people during the period spanning from 2015 to 2018 in the US [22]. Another study using suicide data from the National Violent Death Reporting System spanning from 2005 to 2017 has demonstrated that Medicaid expansion can help mitigate the rise in suicide rate, particularly in non-elderly adults [21]. In this study, we observed results consistent with previous findings, showing a significant decrease in SHVR as Medicaid enrollment rate and Medicaid spending per enrollee increased, with a more pronounced effect in AYA.

Pellegrini and Rodriguez-Monguio have reported that an increase of Medicaid enrollment rate is significantly associated with employment of psychologists and that an increase of employment of psychologists could attenuate suicide rates [23]. Medicaid enrollment has increased almost 5% since the expansion after ACA in US with lack of healthcare insurance decreased by 6.8% in patients with mental illness and addictive disorders [26]. We found that an increase of 1,000 Korean Won in Medicaid spending per enrollee from government was associated with an approximate 1% decrease in SHVR. This finding seems consistent with previous study by Pellegrini and Rodriguez-Monguio and another study by Saloner [23, 26]. Medicaid enrollment numbers of 1,564,725 (3.08%), 1,544,267 (3.03%), 1,509,472 (2.95%), 1,485,740 (2.90%) and 1,484,671 (2.89%) observed from 2014 to 2018 according to the national statistical survey in South Korea suggested a downward trend in the percentage of the population covered by Medicaid. SHVR in the population over 14 years old has increased during the same period from 2014 to 2018, with values of 23,254 (45.6 per 100,000), 23,637 (46.4), 24,807 (48.5), 24,807 (57.4) and 32,745 (63.8) [11].

This research analyzed associations of SHVR with Medicaid coverage variables in a general population older than 14 years. Concurrently, we performed an analysis specifically targeting AYA aged 15–34 years who had the largest percentage of self-harm cases. We also explored a sub-population comprising individuals aged 60 and over. The suicide rate in this sub-population reached a peak of 500 per 100,000 annually across all age groups according to suicide statistics in Korea [11, 27]. When Medicaid enrollment rate was increased by 1%, there was a 4% reduction in SHVR across the general population older than 14 years in this study. Notably, when focusing specifically on the AYA, this reduction was amplified to 14%. Patel et al. have examined connections between Medicaid expansion and specifically reported a reduction in suicide rate among young adults aged 20 to 29 years old [22]. Similarly, Austin et al. have reported a notable reduction in suicide rate among young adults aged 30 to 44 years as a result of Medicaid expansion [21]. Our study uncovered similar results regarding the decrease in SHVR with a simultaneous increase in Medicaid enrollment rate in the population of those aged 15–34 years. This could be attributed to the fact that younger individuals are often less prone to having pre-existing chronic medical conditions and are more likely to seek mental health care. Patel et al. have proposed that ACA expansion, which is linked to better access to mental health care, could substantially reduce suicide rates, aligning with our findings [22].

Conversely, a prior study has revealed minimal expansion-associated reduction of suicide rate in women and older adults, aligning with findings of the present study showing weaker changes of VRR in female and older adults [22]. This observation indicates that factors such as disability and social discrimination known to hinder access to mental health services are more evident in women and older people. It indicates that current efforts to remove such discrimination and obstacles should be continued.

In a quasi-experimental study conducted to assess effects of Medicaid expansion in California, it was found that expansion of Medicaid coverage was linked to a reduction of 0.45 psychiatric ED visits per 1,000 individuals among those aged 19–25 years [28]. However, in our study, it was found that for every 1% increase in Medicaid enrollment rate, there was a decrease of approximately 9.36 individuals with self-harm-related visits per 100,000 young people. This relatively weak impact on the visit rate might be attributed to the nature of this study focusing on visits after self-harm rather than visits resulting from overall psychiatric issues.

Previous studies focusing on the relationship between Medicaid expansion and suicide rates have demonstrated noteworthy decreases in suicide rate among a younger population after expanding Medicaid services [21, 22, 29]. Austin et al. have reported that reductions of suicide rates within states that have expanded Medicaid are more pronounced among male population at a rate of -1.9 per 100,000 compared to female population at a rate of -1.5 per 100,000 [21]. Findings of the present study revealed a consistent pattern in which the decline in SHVR corresponded with an increase in Medicaid enrollment rate across all age categories except for the older female group. Additionally, it was worth noting that R-squared value was significantly greater for the male population than for the female population. This suggests that Medicaid coverage expansion establishes a stronger connection with SHVR for the young adults population, aligning with results obtained findings from the above cited studies.

To prevent enrollment omissions and improve guarantees and assurances for enrollees, South Korean Government has been implementing Medicaid case management service since 2003. The provision of Medicaid case management services is handled by approximately 630 Medicaid case managers across 226 cities, counties, and districts [30]. These managers are mainly comprised of nurses, with a few social workers. Medical assistance case management aims to discover new recipients, screen for high-risk recipients, and find those who require intensive case management. Medical assistance managers are responsible for tasks such as informing recipients about the medical assistance system, consulting on the use of medical institutions, and providing health education and counseling through visits, phone calls, letters, and resource connections [30]. There was a consistent decrease in Medicaid enrollment rate, coupled with a simultaneous increase in SHVR throughout the duration of this study. This emphasizes the importance of initiatives focusing on identifying and supporting high-risk populations within the community to enable them to access Medicaid benefits. Such efforts are essential for addressing the utilization of EDs resulting from self-harm.

Research on the use of psychiatric ED by Medicare enrollees after ACA expansion has indicated that coverage expansion is linked to a significantly decelerated rate of growth in ED utilization with any psychiatric diagnosis [28]. Expansion of Medicaid coverage was associated with a decrease of 0.45 psychiatric ED visits per 1,000 individuals, especially in California. This finding highlights disparities between before and after Medicaid expansion. Conversely, our study demonstrated that expanding Medicaid coverage under consistent eligibility criteria within a single country could positively impact mental well-being and self-harm prevention of community members. This indicates the need for not only broadening legal standards for Medicaid coverage, but also for identifying and providing special support for individuals with limited access to such a social welfare system, such as those with disabilities. However, in this study, it was found that an increase in annual Medicaid visits per enrollee was associated with an increase in SHVR. The likely cause of this trend might be increased prevalence of chronic, incurable medical conditions such as psychiatric illness, renal failure and cancer, which might have resulted in an upsurge of Medicaid enrollment.

This study has some limitations. We conducted an analysis of data from self-harm patients seeking medical attention in ED between 2014 and 2019, which represented a relatively brief study period. However, we endeavored to address this limitation by conducting a comparative analysis encompassing data from 14 distinct provinces. Furthermore, it was noteworthy that this study relied on COVID-19 pandemic data predating the year 2020, which could be considered outdated. Due to the COVID-19 pandemic, there were significant changes in the occurrence of self-harm related ED visits. Thus, if data from after the COVID-19 pandemic were analyzed, different results from ours might be obtained [2]. The third limitation was associated with the fact that this study focused solely on self-harm patients who attended EDs, excluding those with minor self-harm injuries treated at outpatient clinics and patients who did not seek medical care. It is necessary to conduct a thorough investigation into the impact of Medicaid coverage by encompassing patients who engage in mild self-harm.

## Conclusions

This study, which employs nationwide longitudinal ED surveillance data indicates that expanding Medicaid coverage is linked to a reduction in the rate of ED visits after self- harm. These findings suggest that offering a social safety net to underserved socio-economic groups and enabling them to access essential medical and psychological cares can help alleviate overcrowding in EDs by reducing visits after self-harm. Hence, vigorous efforts advocating expansion and improved access to the social welfare and Medicaid system should be continued.

## Supporting information

S1 Fig Histograms show frequency distribution of emergency department self-harm visit rate. The height of each bar in the histogram is visit rate per 100,000 after self-harm in each year. Primary outcome parameter of this study is the self-harm visit rate standardized by population size, which is subsequently aligned with the Poisson distribution.
(PDF)

S1 Table Summary of variables associated with Medicaid coverage during research period (Data are presented as median values and interquartile range). (PDF)

S2 Table Demographic profiles of overall self-harm patients over each year. (PDF)

S3 Table Demographic characteristics of self-harm patients categorized by age groups. (PDF)

S4 Table Results of multicollinearity test. (PDF)

S1 Text Extended results. (PDF)

## References

[1] World Health Organization. Suicide worldwide in 2019: global health estimates. 2021.

[2] Lee YJ, Yuh MA, Kim IS, Cho BNH, Woo SH, Hong S. Physical distancing and emergency medical services utilization after self-harm in Korea during the early COVID-19 pandemic: A nationwide quantitative study. PLoS one. 2023;18(5):e0286398. 10.1371/journal.pone.0286398. 37252929 PMC10228815

[3] Romero-Pimentel AL, Mendoza-Morales RC, Fresan A, Garcia-Dolores F, Gonzalez-Saenz EE, Morales-Marin ME, et al. Demographic and clinical characteristics of completed suicides in Mexico City 2014–2015. Frontiers in psychiatry. 2018;9:402. 30245640 10.3389/fpsyt.2018.00402 PMC6137233

[4] Hawton K. Deliberate self-harm in Oxford 1996: Oxford University Department of Psychiatry; 1996.

[5] Hawton K, Zahl D, Weatherall R. Suicide following deliberate self-harm: long-term follow-up of patients who presented to a general hospital. British Journal of Psychiatry. 2003;182:537–42. Epub 2003/06/05. 10.1192/bjp.182.6.537. 12777346

[6] Griffin E, McMahon E, McNicholas F, Corcoran P, Perry IJ, Arensman E. Increasing rates of self-harm among children, adolescents and young adults: a 10-year national registry study 2007–2016. Social psychiatry and psychiatric epidemiology. 2018;53:663–71. 10.1007/s00127-018-1522-1. 29721594

[7] Brathwaite D, Waller AE, Gaynes B, Deselm TM, Bischof JJ, Tintinalli J, et al. Age and sex trends among mental health-related emergency department visits in North Carolina. Healthcare Analytics. 2022;2:100056. 10.1016/j.health.2022.100056.

[8] Hawton K, Harriss L. Deliberate self‐harm in people aged 60 years and over: characteristics and outcome of a 20‐year cohort. International Journal of Geriatric Psychiatry: A journal of the psychiatry of late life and allied sciences. 2006;21(6):572–81. 10.1002/gps.1526. 16645937

[9] Troya MI, Babatunde O, Polidano K, Bartlam B, McCloskey E, Dikomitis L, et al. Self-harm in older adults: systematic review. The British Journal of Psychiatry. 2019;214(4):186–200. 10.1192/bjp.2019.11. 30789112

[10] Curtis C. Female deliberate self-harm: the women’s perspectives. Women’s Studies. 2018;47(8):845–67. 10.1080/00497878.2018.1524762.

[11] Gong AK, Yun JH, Kim IS, Yuh MA, Woo SH, Kim J, et al. Factors affecting emergency medical utilization after self-harm and effectiveness of community-based suicide prevention provisions in preventing self-harm: a nationwide registry-based study in Korea. Community Mental Health Journal. 2023;59:942–53. 10.1007/s10597-022-01077-8. 36547814 PMC9772591

[12] Hamermesh DS, Soss NM. An economic theory of suicide. Journal of Political Economy. 1974;82(1):83–98. 10.1086/260171.

[13] Ying Y-h, Chang K. A study of suicide and socioeconomic factors. Suicide and Life-threatening Behavior. 2009;39(2):214–26. 10.1521/suli.2009.39.2.214. 19527162

[14] Simon K, Soni A, Cawley J. The impact of health insurance on preventive care and health behaviors: evidence from the first two years of the ACA Medicaid expansions. Journal of Policy Analysis and Management. 2017;36(2):390–417. 10.1002/pam.21972. 28378959

[15] Donohue JM, Cole ES, James CV, Jarlenski M, Michener JD, Roberts ET. The US Medicaid program: coverage, financing, reforms, and implications for health equity. Journal of the American Medical Association. 2022;328(11):1085–99. 10.1001/jama.2022.14791. 36125468

[16] Ministry of Health and Welfare. Introduction for medical aid program in 2010. Ministry Health and Welfare 2009. p. 401.

[17] Ministry of Health and Welfare. Introduction for medical aid program in 2023. Ministry Health and Welfare; 2022. p. 671.

[18] Fry CE, Sommers BD. Effect of Medicaid expansion on health insurance coverage and access to care among adults with depression. Psychiatric Services. 2018;69(11):1146–52. 10.1176/appi.ps.201800181. 30152271 PMC6395562

[19] Winkelman TN, Chang VW. Medicaid expansion, mental health, and access to care among childless adults with and without chronic conditions. Journal of General Internal Medicine. 2018;33:376–83. doi: 10.1007/s11606-017-4217-5 29181792 PMC5834959

[20] McMorrow S, Gates JA, Long SK, Kenney GM. Medicaid expansion increased coverage, improved affordability, and reduced psychological distress for low-income parents. Health Affairs. 2017;36(5):808–18. 10.1377/hlthaff.2016.1650. 28461346

[21] Austin AE, Naumann RB, Short NA. Association between Medicaid expansion and suicide mortality among nonelderly US adults. American Journal of Epidemiology. 2021;190(9):1760–9. doi: 10.1093/aje/kwab130 34467410 PMC12931419

[22] Patel H, Barnes J, Osazuwa-Peters N, Bierut LJ. Association of state Medicaid expansion status with rates of suicide among US adults. JAMA Network Open. 2022;5(6):e2217228–e. 10.1001/jamanetworkopen.2022.17228. 35704315 PMC9201676

[23] Pellegrini LC, Rodriguez-Monguio R. Unemployment, Medicaid provisions, the mental health industry, and suicide. The Social Science Journal. 2013;50(4):482–90. 10.1016/j.soscij.2013.09.013.

[24] Akoglu H. User’s guide to correlation coefficients. Turkish Journal of Emergency Medicine. 2018;18(3):91–3. doi: 10.1016/j.tjem.2018.08.001 30191186 PMC6107969

[25] Hawton K, Casañas i Comabella C, Haw C, Saunders K. Risk factors for suicide in individuals with depression: a systematic review. Journal of Affective Disorders. 2013;147(1–3):17–28. 10.1016/j.jad.2013.01.004. 23411024

[26] Saloner B. An update on “Insurance coverage and treatment use under the Affordable Care Act among adults with mental and substance use disorders”. Psychiatric Services. 2017;68(3):310–1. 10.1176/appi.ps.201600566. 28240146

[27] Statistics Korea. Annual report on the causes of death statistics; 2020 2020 [14 February 2022]. Available from: https://kostat.go.kr/portal/eng/pressReleases/1/index.board?bmode=read&aSeq=414516.

[28] Golberstein E, Busch SH, Zaha R, Greenfield SF, Beardslee WR, Meara E. Effect of the Affordable Care Act’s young adult insurance expansions on hospital-based mental health care. American Journal of Psychiatry. 2015;172(2):182–9. 10.1176/appi.ajp.2014.14030375. 25263817 PMC4314328

[29] Barnes JM, Graboyes EM, Adjei Boakye E, Kent EE, Scherrer JF, Park EM, et al. The Affordable Care Act and suicide incidence among adults with cancer. Journal of Cancer Survivorship. 2023;17(2):449–59. doi: 10.1007/s11764-022-01205-z 35368225

[30] Park EJ, Kim C. Case management process identified from experience of nurse case managers. Journal of Korean Academy of Nursing. 2008;38(6):789–801. doi: 10.4040/jkan.2008.38.6.789 19122481

