# Supplementary material for: Association of Medicaid coverage with emergency department utilization after self-harm in Korea: A nationwide registry-based study
Source: PLoS One. 2024 Jun 25;19(6):e0306047. doi: 10.1371/journal.pone.0306047 (PMC11198744; doi:10.1371/journal.pone.0306047)
Supplement: S1 Fig — The height of each bar in the histogram is visit rate per 100,000 after self-harm in each year. Primary outcome parameter of this study is the self-harm visit rate standardized by population size, which is subsequently aligned with the Poisson distribution. (PDF) [file pone.0306047.s001.pdf]

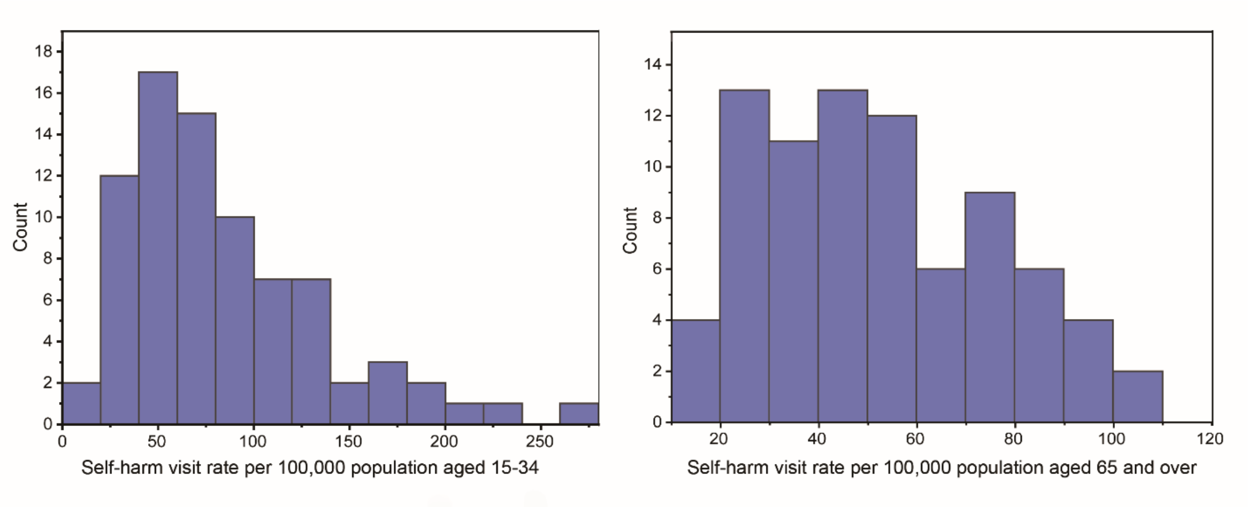

S1 Fig. Histograms show frequency distribution of emergency department self-harm visit rate. The height of each bar in the histogram is visit rate per 100,000 after self-harm in each year. Primary outcome parameter of this study is the self-harm visit rate standardized by population size, which is subsequently aligned with the Poisson distribution.
