# Supplementary material for: Association of Medicaid coverage with emergency department utilization after self-harm in Korea: A nationwide registry-based study
Source: PLoS One. 2024 Jun 25;19(6):e0306047. doi: 10.1371/journal.pone.0306047 (PMC11198744; doi:10.1371/journal.pone.0306047)
Supplement: S1 Table — (PDF) [file pone.0306047.s002.pdf]

S1 Table. Summary of variables associated with Medicaid coverage during research period (Data are expressed as median values and interquartile range).

Variable	2014	2015	2016	2017	2018	2019
Medicaid enrollee	83,439.8 (56,588.0, 99889.2)	86,020.4 (58,338.2, 102978.6)	87,034.7 (57810.0, 101318.5)	86,643.5 (57673.1, 99238.1)	83,822.8 (56745.8, 104632.0)	79,840.0 (54377.2, 96605.0)
Medicaid enrollment rate (%)	3.8 (3.2, 4.8)	3.7 (3.1, 4.3)	3.7 (3.1, 4.3)	3.4 (3.0, 4.2)	3.4 (3.0, 4.3)	3.3 (3.0, 4.1)
Medicaid coverage days	4,170,010.0 (2,987,188.0, 6,646,195.0)	4,247,076.2 (2,720,335.8, 5,092,137.0)	4,701,524.0 (2,90,9421.0, 5,788,128.0)	4,371,806.5 (2,864,300.5, 5,053,018.5)	4,601,782.2 (2,878,887.3, 5,395,941.8)	5,035,721.0 (2,910,182.0, 6,993,298.5)
Medicaid coverage days per enrollee	50.1 (47l6, 56.5)	45.9 (42.0, 48.6)	48.7 (43.0, 51.7)	46.9 (44.4, 51.7)	48.5 (45.9, 53.9)	53.2 (50.7, 60.8)
Medicaid visits	4,870,010.2 (2,987,188.0, 6,646195.0)	6,355,641.8 (3,922,715.8, 8,346,565.0)	7,266,692.0 (4,213,426.0, 8,829,792.0)	6,665,897.2 (3,980,537.5, 8,326,720.5)	6,843,045.1 (4,052,242.3, 8,803,461.8)	6,904,754.2 (4,629,361, 8,839,033.6
Medicaid visits per enrollee	59.4 (55.6, 66.0)	68.8 (62.9, 74.8)	75.4 (66.5, 79.8)	71.5 (67.7, 78.9)	72.2 (68.2, 80.2)	72.9 (69.5, 83.2)
Admission days per enrollee	4.7 (4.6, 5.1)	4.8 (4.5, 5.2)	4.8 (4.6, 5.3)	5.1 (4.7, 5.40)	5.2 (4.9, 5.6)	5.3 (5.0, 5.6)
Crude Medicaid spend (1,000,000 Korea Won)	461709.0 (288388.9, 575517.9)	434290.4 (266828.6, 542729.7)	383051.3 (235848.8, 477481.9)	403567.0 (243995.0, 492742.2)	335224.9 (212461.1, 438362.7)	325168.2 (206087.2, 425211.8)
Medicaid spend per enrollee (1,000 Korea Won)	5,548.9 (5,291.0, 5,900.6)	4,880.9 (4,641.7, 5,213.2)	4,250.1 (4,041.7.4,708.7)	4,458.1 (4,246.8, 4,954.4)	3,812.7 (3,648.1, 4,177.4)	3,812.7 (3,648.2, 4,177.5)
