# Supplementary material for: Association of Medicaid coverage with emergency department utilization after self-harm in Korea: A nationwide registry-based study
Source: PLoS One. 2024 Jun 25;19(6):e0306047. doi: 10.1371/journal.pone.0306047 (PMC11198744; doi:10.1371/journal.pone.0306047)
Supplement: S2 Table — (PDF) [file pone.0306047.s003.pdf]

S2 Table. Demographic profiles of overall self-harm patients over each year.

Variable		2014	2015	2016	2017	2018	2019	(Cramer 's V) P-value
Gender, n (%)	Female	11,798 (54.0)	12,595 (54.2)	12,905 (54.6)	13,807 (55.7)	16,497 (56.1)	18,503 (56.5)	(0.023) 0.731
	Male	10,065 (46.0)	10,659 (45.8)	10,732 (45.4)	11,000 (44.3)	12,920 (43.9)	14,237 (43.5)	
Age, n (%)	15-24	4,115 (18.8)	4,405 (18.9)	4,591 (19.4)	5,307 (21.4)	7,216 (24.5)	8,122 (24.8)	(0.059) 0.044
	25-34	4,636 (19.1)	4,685 (18.4)	4,796 (18.5)	4,784 (18.4)	5,062 (17.7)	5,732 (17.5)	
	35-44	4,266 (21.2)	4,460 (20.1)	4,371 (19.9)	4,500 (19.3)	5,165 (17.2)	5,436 (16.6)	
	45-54	4,167 (19.5)	4,271 (19.2)	4,364 (18.6)	4,564 (18.1)	5,205 (17.6)	5,731 (17.5)	
	55-64	2,118 (9.7)	2,506 (10.8)	2,612 (11.2)	2,652 (10.7)	3,307 (11.2)	3,897 (11.9)	
	65-74	1,331 (6.1)	1,467 (6.3)	1,417 (6.7)	1,403 (5.7)	1,534 (5.2)	1,633 (4.99)	
	75-84	634 (5.1)	719 (5.0)	780 (5.7)	863 (5.0)	1,024 (5.2)	1,767 (5.4)	
	85-94	588 (1.4)	719 (1.2)	685 (1.5)	718 (1.4)	880 (1.3)	425 (1.3)	
	95-	8 (0.0)	22 (0.0)	21 (0.0)	16 (0.0)	24 (0.0)	3 (0.0)	
Self-harm method, n (%)	Poisoning	12,871 (58.9)	15,507 (58.9)	15,442 (57.8)	15,790 (56.8)	18,770 (57.5)	18,861 (57.6)	(0.124) 0.023
	Stabbing	4,351 (19.9)	5,316 (21.3)	5,779 (22.9)	6,396 (24.4)	7,488 (24.1)	7,761 (23.7)	
	Chocking	1,815 (8.3)	1,715 (7.4)	1,683 (7.1)	1,722 (6.9)	2,021 (6.9)	2,227 (6.8)	
	Struck	940 (4.3)	1,131 (4.3)	1,172 (4.4)	1,129 (4.1)	1,271 (3.9)	1,343 (4.1)	
	Fall	547 (2.5)	621 (2.7)	623 (2.6)	578 (2.3)	749 (2.5)	786 (2.4)	
	Submersion	262 (1.2)	292 (1.3)	273 (1.2)	282 (1.1)	313 (1.1)	392 (1.2)	
	Burn	109 (0.4)	80 (0.3)	76 (0.3)	61 (0.2)	73 (0.2)	131 (0.4)	
	TA	87 (0.4)	97 (0.4)	76 (0.3)	68 (0.3)	85 (0.3)	98 (0.3)	
	Machine	22 (0.1)	21 (0.1)	6 (0.0)	7 (0.0)	8 (0.0)	33 (0.1)	
	Other	721 (3.3)	768 (3.3)	732 (3.1)	847 (3.4)	867 (2.9)	949 (2.9)	
	Unknown	138 (0.6)	126 (0.5)	158 (0.6)	108 (0.4)	215 (0.7)	164 (0.5)	
Total		21,863	23,254	23,637	24,807	29,417	32,745	

TA, Traffic accident.
