# Supplementary material for: Association of Medicaid coverage with emergency department utilization after self-harm in Korea: A nationwide registry-based study
Source: PLoS One. 2024 Jun 25;19(6):e0306047. doi: 10.1371/journal.pone.0306047 (PMC11198744; doi:10.1371/journal.pone.0306047)
Supplement: S3 Table — (PDF) [file pone.0306047.s004.pdf]

S3 Table. Demographic characteristics of self-harm patients categorized by age groups.

Variable		Younger (15-34)	Mid-age (35-64)	Older (> 65)	(Cramer's V) P-value
Year, n (%)	2014	8,981 (13.3)	8,465 (14.7)	4,215 (13.7)	(0.041) < 0.001
	2015	9,365 (13.9)	8,925 (15.5)	4,815 (15.7)	
	2016	9,706 (14.4)	8,983 (15.6)	4,933 (16.1)	
	2017	10,657 (15.8)	9,213 (16.0)	4,872 (15.9)	
	2018	13,389 (19.9)	1,402 (18.3)	5,702 (18.6)	
	2019	15,310 (22.7)	11,459 (19.9)	6,192 (20.2)	
Gender, n (%)	Female	29,322 (43.5)	38,714 (67.2)	18,069 (58.8)	(0.207) < 0.001
	Male	38,086 (56.5)	18,872 (32.8)	12,660 (41.2)	
Self-harm method, n (%)	Poisoning	42,938 (63.7)	34,118 (59.2)	22,831 (74.3)	(0.637) < 0.001
	Chocking/hanging	4,785 (7.1)	12,956 (22.5)	3,226 (10.5)	
	Stabbing	4,448 (6.6)	3,915 (6.8)	2,212 (7.2)	
	Struck by object	6,336 (9.4)	2,418 (4.2)	706 (2.3)	
	Fall	3,100 (4.6)	1,402 (2.4)	834 (2.7)	
	Drowning	1,230 (1.8)	633 (1.1)	257 (0.8)	
	TA	337 (0.5)	188 (0.3)	97 (0.3)	
	Burn	252 (0.3)	172 (0.3)	65 (0.2)	
	Machine	67 (0.1)	57 (0.1)	6 (0.0)	
	Other	3,640 (5.4)	17,42 (3.0)	399 (1.3)	
	Unknown	275 (0.4)	909 (0.6)	96 (0.3)	
Mental status, n (%)	Alert	32,086 (47.6)	35,242 (61.2)	24,460 (79.6)	(0.201) <0.001
	Verbal	13,751 (20.4)	9,962 (17.3)	3,472 (11.3)	
	Pain	12,133 (18.0)	8,407 (14.6)	1659 (5.4)	
	Unresponsive	9,438 (14.0)	3,973 (6.9)	1138 (3.7)	
Total		67,408 (100.0)	57,586 (100)	30,729 (100.0)	

TA, Traffic accident.
