# Supplementary material for: Association of Medicaid coverage with emergency department utilization after self-harm in Korea: A nationwide registry-based study
Source: PLoS One. 2024 Jun 25;19(6):e0306047. doi: 10.1371/journal.pone.0306047 (PMC11198744; doi:10.1371/journal.pone.0306047)
Supplement: S4 Table — (PDF) [file pone.0306047.s005.pdf]

S4 table. Results of multicollinearity test.

Population group	Dependent variable	Independent variable	R-squared	VIF
Entire adults	SHVR	Medicaid enrollment rate	0.114	1.129
		Medicaid spending per enrollee	0.109	1.123
		Medicaid visits	0.198	1.247
		Hospital stays per enrollee	0.108	1.122
Older adults	SHVR	Medicaid enrollment rate	0.607	2.548
		Medicaid spending per enrollee	0.179	1.219
		Medicaid visits	0.254	1.341
		Hospital stays per enrollee	0.388	1.636
Young adults	SHVR	Medicaid enrollment rate	0.194	1.242
		Medicaid spending per enrollee	0.474	1.902
		Medicaid visits	0.176	1.215
		Hospital stays per enrollee	0.296	1.421

SHVR; self-harm visit rate, VIF; variance inflation factor.

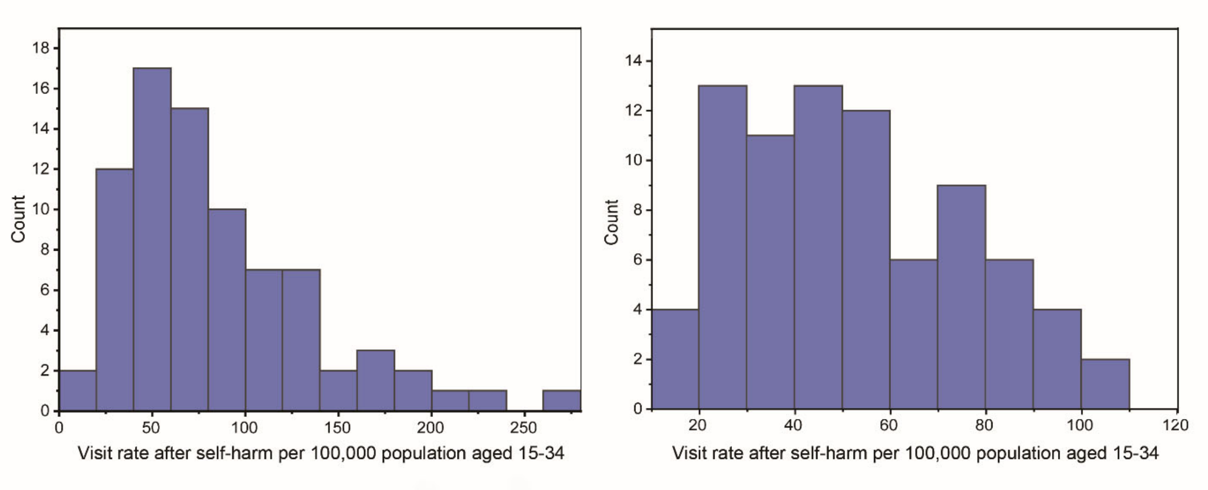

Supplementary Fig. 1. Histograms show the frequency distribution of emergency department self-harm visit rate. The height of each bar in the histogram is visits rate per 100,000 after self-harm in each year. The primary outcome parameter of this study is the visit rate after self-harm standardized by population, which is then matched to the Poisson distribution.
