# Supplementary material for: Association of Medicaid coverage with emergency department utilization after self-harm in Korea: A nationwide registry-based study
Source: PLoS One. 2024 Jun 25;19(6):e0306047. doi: 10.1371/journal.pone.0306047 (PMC11198744; doi:10.1371/journal.pone.0306047)
Supplement: S1 Text — (PDF) [file pone.0306047.s006.pdf]

S1 Text. Extended results for

"Association of Medicaid coverage with emergency medical service utilization after self-harm in Korea: A nationwide registry-based study"

Ga In Han, Sikyoung Jeong, Insoo Kim, Min Ah Yuh, Seon Hee Woo, and Sungyoup Hong

A total of 186,731 patients aged 14 years and older visited emergency departments (EDs) seeking medical attention for self-inflicted injury or poisoning from January 2014 to December 2019. Of them, 1,245 patients had an unclear onset of self-harm. In addition, 19,748 were excluded because they had been transferred from other medical facilities. Additionally, 10,015 patients were removed from the research sample due to expiration either upon arrival at the ED or during their treatment in the ED. The remaining 155,723 patients were finally included in this study. S2 Table offers a comprehensive summary of demographic and clinical features of these self-harm patients categorized by years. The sex ratio of men to women remained constant over time ($p = 0.731$). Regarding the demographic distribution of self-harm patients, there was a significant rise in the 15-24 age category throughout the study, whereas a decline was observed in the remaining age groups (Cramer's V=0.059, $p = 0.044$). Analysis of self-harm methods revealed a decline in incidents of poisoning, choking, hanging, being struck by objects, and falls, while incidents of stabbing were on the rise (V = 0.124, $p = 0.023$).

Of the 155,723 patients who sought ED care due to self-harm, 67,408 were younger adults (aged 15 to 34), 30,729 were older adults (aged 65 and older), and 57,586 were middle-aged (aged 45 to 64) (S3 Table). Females constituted 86,105 patients, representing 55.2% of the total. In the older age group, women made up 58.8%, while in the younger age group, they constituted 43.5%. There was a clear linear relationship in which the proportion of women increased with

age (V=0.207, p < 0.001). Poisoning was the predominant method of self-harm in both age groups, accounting for 57.9% of cases, followed by choking/hanging, stabbing, and being struck by an object. Linear association analysis of self-harm method according to age showed a very strong correlation with increasing age (V = 0.637, $p < 0.001$). Poisoning increased with increasing age, whereas falls, being struck by an object, drowning, and traffic accidents showed decreases with increasing age. A total of 56,546 (57.6%) self-harm patients who visited EDs were alert upon arrival. The percentage of alert patients was notably higher in older adults compared to other age groups (V = 0.201, $p < 0.001$).
